# Is Rejection, Parental Abandonment or Neglect a Trigger for Higher Perceived Shame and Guilt in Adolescents?

**DOI:** 10.3390/healthcare11121724

**Published:** 2023-06-12

**Authors:** Marius Marici, Otilia Clipa, Remus Runcan, Loredana Pîrghie

**Affiliations:** 1 Faculty of Educational Sciences, Stefan cel Mare University, 720229 Suceava, Romania; 2 Faculty of Educational Sciences, Psychology and Social Work, Aurel Vlaicu University of Arad, 310032 Arad, Romania; remus.runcan@uav.ro

**Keywords:** abandonment, shame, guilt, parental rejection, adolescents and teenagers

## Abstract

Theories of development point out that childhood experiences are relevant across the lifespan, and that the parent-child relationship is essential for a child’s physical and psychological wellbeing. The aim of this study is to investigate whether parental abandonment influences self-conscious emotions such as guilt and shame. This quasi-experiment included 230 adolescents and teenagers (M = 17.1, SD = 1.82), and data were collected via a self-reported questionnaire administered online. We used the Guilt Inventory, the Experience of Shame Scale, the Childhood Trauma Questionnaire, and the Parental Acceptance/Rejection Questionnaire. Results indicated that the child’s environment was significantly associated with feelings of shame. Abuse is associated with both guilt and shame, while paternal rejection is associated with guilt. The environment in which children and teenagers develop is associated with how they perceive themselves in relation to others. This study underlines the importance of considering child development conditions and the paramount importance of social work assistance for abandoned children and teenagers.

## 1. Introduction

The abandonment of children is a heartbreaking reality that affects countless lives. It is a problem that requires a multifaceted approach, involving not only government agencies but also community organizations and individuals. As a society, we have a moral, ethical, political, and legal obligation to protect our children and provide resources and support to families in need so that they can provide a stable and loving home for children [1]. Adverse childhood experiences, feelings of shame and guilt, and the inability to achieve goals can significantly impact an individual’s trajectory and relationships with others in adulthood. These factors can have a profound and lasting effect on a person’s overall well-being and success [2]. Scientific literature provides little information on the association between child abandonment and self-conscious emotions, usually referring to the relationship between maltreatment and proneness to shame and guilt [3].

## 2. Child Abandonment Concept and Its Consequences

Child abandonment is often related, in scientific literature, with child neglect, child abuse or parental rejection [4].

An abandoned child is a child whose biological parents no longer fulfil their responsibility to care for and to provide, thereby ignoring their basic developmental needs [5,6]. Child abandonment represents the worst form of neglect (Law no. 272/2004, art. 94, paragraph 2). The American Psychological Association associates child neglect with ‘the denial of attention, care or affection considered essential for the normal development of a child’s physical, emotional, and intellectual qualities, usually due to indifference from, disregard by, or impairment in the child’s caregivers’ [7]. In literature, child neglect is associated more with the experience of shame than guilt [8]. Neglect represents a risk factor for children’s physical and psychological well-being. Child abuse can be defined as ‘the deliberate use of force, physically or psychologically to carry out acts that have the possibility of causing developmental disruption as well as injury to the mental and physical state of a child’ [9]. Children, as victims of abuse and neglect, show high levels of shame [8].

Abandonment refers to a situation where a child is intentionally left behind, openly or secretly, by a parent who has no intention of returning. This abandonment is a voluntary relinquishment of parental responsibility, and no other family members are willing or able to take on the role of caregiver. Among various forms of abuse, the loss of one or both parents leads to what literature calls the Abandoned Child Syndrome, a behavioral or psychological condition. Abandonment may be physical (parental absence) or emotional (refusal of the parents to provide affection, care, or stimulation). Abandonment means breaking any bonds, especially emotional ones, with the parent or primary caregiver. According to the findings of another study, institutionalizing children can expose them to a high risk of experiencing trauma or living with trauma. This means that placing children in institutions may not be the best solution for addressing their needs, for it can have negative effects on the children’s mental health and overall well-being [10]. Marshall and Kenney in 2009 [11] have shown that those children who grow up in an institutional environment are at risk of developing physical, emotional, cognitive, behavioral, or social problems. Factors that cause these problems may include negative childhood experiences, age of admission to the institution, the length of time spent there, lack of interaction between children and caregivers, and lack of the presence of a constant caregiver. Alexandrescu in 2021 writes about the suffering of abandoned children resulting from abuse and neglect that can be physical, sexual, and emotional, noting that ‘the main lesson the system teaches them is that they are to blame’ [1] (p. 46) for their institutionalization. Placing a child with a history of institutionalization in a family environment that responds to his or her needs is linked to developmental improvements at all levels [11]. The mother/caregiver’s inability to show affection to the child has the strongest negative impact on the child’s development, making children feel rejected and unaccepted. The lack of maternal responsiveness, a ‘high risk factor’ [12] for a child’s well-being, is characterized by difficulties in identifying and responding to the child’s emotional needs or disruptions in supporting the child’s autonomy. This leads to a higher level of child vulnerability and to negative consequences regarding child externalization [12]. 

Parental rejection is a highly traumatic experience for a child, as it implies a lack of interest and affection from the parent or caregiver. This can lead to both physical and psychological damage, as the child may feel unloved and unwanted [13,14,15]. It is crucial for parents to express their feelings of love and support towards their children in healthy and helpful ways, as this can have a significant impact on their overall well-being and development. Failure to do so can result in long-lasting emotional scars that may affect the child’s relationships and self-esteem in the future. According to the Acceptance-Rejection Theory [16], parental rejection negatively affects both the child’s psychological and behavioral adjustment. The negative consequences of parental rejection [17] and institutionalization [18] are also supported by studies in the literature [19].

## 3. The Association of Shame or Guilt with Parental Abandonment

The literature on shame and guilt refers to these as ‘self-conscious emotions’ [20]. Usually studied together, these emotions differ in how the self is evaluated. ‘The experience of shame is directly about the self which is the focus of evaluation. In guilt, the self is not the central object of negative evaluation, but rather the thing done or undone is the focus. In guilt, the self is negatively evaluated in connection with something but is not itself the focus of the experience’ [21]. Shame, recognized as a negative emotion, involves an inferiority complex, powerlessness, self-consciousness, and a desire to hide flaws [20]. Shame is often seen as a decreased need for affection from parents, as a tendency to be more egocentric, or as social avoidance [22,23].

Parental rejection was positively related to self-conscious emotions [24].

Abandonment involves rejection, and the loss of the other’s love is a self-shaming experience [25,26]. Being raised in an institutionalized environment is associated with being stigmatized, which can lead to feelings of shame. Another study notes that shame is often associated with fear of being rejected and abandoned, with behaviors of isolation and submission. All these reinforce the children’s ideas that that they are despised [20].

The literature documents the idea that parental rejection is associated directly with shame in children [27]. It is evident that children who do not receive affection and love from their parents may develop a belief that they are undeserving of such emotions. This can lead to a sense of inadequacy and a feeling of being “defective”. Ultimately, these negative emotions can manifest as feelings of shame [28]. Guilt is felt less intensely than shame. Guilt lasts a shorter time and is less debilitating than shame. However, this does not exempt the detrimental impact of guilt on children who have been abandoned [2]. What is more, adolescents rejected by parents are more likely to experience guilt and are more vulnerable to shame than other children [3,29]. 

Different forms of abuse and neglect might be intertwined with shame and guilt, suggesting that they occur because of parental abuse, neglect or rejection [24]. Children who experience shame resulting from abuse (i.e., physical or sexual) perceive themselves as ‘bad’ (p. 368) in terms of their personal value [20]. Emotional neglect and abuse seem to be positively associated with shame, and less so with feelings of guilt [30]. The findings are inconclusive about the correlation between shame, guilt, and physical neglect. Moreover, the number of studies conducted on this topic is limited. [30]. In contrast, the results of another study [31] showed that all types of severe violence, childhood sexual abuse, exposure to violence, and physical abuse are positively associated with feelings of shame and guilt, while noting that the greater the number of negative experiences, the more intensely these emotions are felt. 

The child who experiences abandonment feels unwanted, rejected by his or her own parents, and deals with feelings of worthlessness that cause shame [32]. 

Unpleasant experiences can significantly alter children’s perception of their relationships with others, leading to a loss of trust in people. This can have a profound impact on their emotional and social development, potentially hindering their ability to form healthy connections with others in the future [2,33]. The relationship between a caregiver and a child can significantly impact the child’s self-perception and ability to handle stressors in life [27]. According to the Attachment Theory, the emotional bond between the mother or the primary caregiver and the child lays the foundation for future relationships in adulthood. A child with insecure attachment exhibits feelings of non-acceptance and experiences difficulties in establishing interpersonal relationships. Thus, all these factors increase the likelihood of developing more intense self-conscious emotions [34]. One of the most pronounced effects of growing up in an institutional environment is attachment disorders due to frequent shifts, many caregivers, and the limited availability of caregivers for emotional sharing.

## 4. Shame and Guilt in Adolescents

Adolescence is a stage when major biological, social and cognitive changes occur [35], a stage prone to vulnerability. Neuropsychological studies have shown that this age is a critical period for processing sociocultural information [36,37,38,39,40]. It is a time of exploration, of abandoning childhood status and beginning adult life. Adolescence is a crucial developmental stage for individuals, regardless of their risk status. This transitional phase can potentially lead to perilous circumstances, resulting in self-estrangement. However, it can also present a favorable opportunity for individuals to comprehend their past traumas and attribute novel significance to them, thereby promoting emotional healing and resilience [41].

In scientific literature, research on shame and guilt frequently focuses on childhood experiences [3], with comparatively less attention paid to adolescence. This stage is characterized by an amplified emotional reactivity to the social environment [3]. During adolescence, sensitivity to self-conscious emotional stimuli is greater than among children and adults [3]. Shame and guilt, manifested as humiliation and worthlessness, can trigger feelings of helplessness and hopelessness, thus leading to an increased risk of suicide, especially during adolescence [30].

Feelings of guilt in adolescence may take adaptive or maladaptive forms [3]. Predispositions to guilt might increase if there is a history of trauma [35]. Guilt is often associated with negative internal and interpersonal consequences [2,42,43].

Feelings of shame can be associated with PTSD, obsessive-compulsive disorder, psychoticism, or depression [3]. Shame can lead to narcissism [44] caused by lack of parental affection or dysfunctional parenting. Lack of parental love, feelings of worthlessness, rejection, and abuse experienced because of abandonment lead to heightened feelings of shame and guilt, and by implication, cause psychological distress [16,45]. Feeling unloved can lead to a sense of total rejection, and this is accompanied by an increased sensitivity to shame, which can set the stage for severe pathology [16]. 

While some may view shame and guilt negatively, others see them in a more positive light. Shame, for example, is often considered a self-focused emotion that is related to competitive behaviors. It can also express the need to prove oneself desirable to others. Guilt, on the other hand, is often associated with a desire to avoid causing harm to others [46]. This is a counterpoint to the classical theories already cited.

Studying emotions like guilt and shame in adolescence might help one to understand the psychological problems and their implications with the aim of improving abandoned children’s normal psychological functioning [16].

## 5. Methodology

### 5.1. The Present Study

The aim of this study is to explore whether abandonment plays a role in the development of self-conscious emotions, specifically guilt and shame, among children and teenagers (14–20 years range).

### 5.2. Procedure

The present research is a quasi-experiment based on a self-reported online questionnaire. Informed consent was obtained from all the participants. They were assured that the data were confidential, to be used strictly for research purposes, and that they could withdraw at any time. 

### 5.3. Hypotheses

The hypotheses of this study are:

**H1.**  *Children and teenagers with higher levels of parental rejection will report stronger feelings of guilt than those with lower levels of rejection.*

**H2.**  *Children and teenagers with higher levels of parental rejection will report stronger feelings of shame than those with lower levels of rejection.*

**H3.**  *Children and teenagers with higher levels of abuse will report more guilt than those with lower levels of abuse.*

**H4.**  *Children and teenagers with higher levels of abuse will report more shame than those with lower levels of abuse.*

**H5.**  *Children and teenagers raised in institutional care will report higher levels of guilt than children raised in a family environment.*

**H6.**  *Children and teenagers raised in institutional care will report higher levels of shame than children raised in a family environment.*

### 5.4. Participants

The questionnaire was distributed to 230 respondents, of whom 68.7% were girls and 32.3% were boys. The participants were secondary and high school students aged between 14 and 20 years (M = 17.1, SD = 1.82). The children and teenagers included in the study were divided into two groups: a group of participants living with their birth or adoptive family and a group of participants living in institutional care. The adoptive family is considered to play the same role and offer the same love and care as the birth family. Of the total number of participants, 42.6% live with both parents, 1.7% live with a relative, 1.7% live with adoptive parents, 3.5% live in foster care, 24.3% live in a placement center, 13.0% live in a ‘family-type home’, 12.2% live with their mother, and 0.9% live with their father. A placement center (‘centru de plasament’) is a residential service in the field of child protection. It was established to provide abandoned children and street children, aged between 7 and 26 years, an opportunity to benefit from optimal physical, intellectual, emotional, and spiritual development. These are the old orphanages, which are now going to be closed because legislation does not encourage them any longer. ‘Foster care’ (‘în plasament familial’) in Romania refers to a place where one or more adults, males/females or a couple who own housing, are capable of bringing up children and playing the role of parental figures for abandoned children. A ‘family-type home’ (‘case de tip rezidențial’) ‘is a dwelling that covers the essential needs for rest, food preparation, education, and hygiene, ensuring the minimum requirements for a maximum of 12 children, for whom the measure of emergency placement or placement has been established, as appropriate, under the present law’ (Law 242/2014, art. 123/4)**.**

Thus, we have created two groups. In the first group, ‘participants living in a family environment’ we included those living with both parents, those living with a mother, those living with a father, those living with a relative, and those living with adoptive parents. In the second group, we included children and teenagers living in residential care, those in family-type households and those living in foster care. 

One difficulty of this study was applying the questionnaire to the group of participants in institutional care, as it was necessary to adapt the questionnaire for the categories of participants living in a home environment and those in institutional settings. The adaptation referred to lexical modifications such as ‘parents’ versus ‘caregivers’, ‘family home’ or ‘institutional care’.

The recruitment of participants was a crucial aspect of our study, and we approached it with a well-planned strategy. We identified key individuals who had access to the participants we needed, such as school principals, coordinators of social assistance centers, and local leaders. Through careful communication and scheduling, we were able to secure appointments with these individuals and visit their institutions to administer the paper questionnaires. However, this process was not without its challenges. The participants we sought were not always easily accessible, and it required persistence and creativity to reach them. All the abandoned participants in our study were from institutions in Suceava. The children and teenagers we studied had been abandoned due to abuse or neglect [47], often because their biological parents were incarcerated, working abroad (a common trend in Romania), or unable to care for them due to unwanted pregnancy, alcoholism, or disorganized families. Our sample included young people of both Romanian or Roma ethnicity. 

In the course of our research, we encountered situations in which subjects were hard to access because institutional officials refused to cooperate with us. We accepted the situation in which subjects responded only to some questions, resulting in missing data within our database. Even though we had 230 respondents in our database, missing data from one variable sometimes did not align with missing data from another variable, particularly where the two variables were associated. As a result, in the final analysis, we had only 70 or 160 cases.

### 5.5. Instruments

The present study used the following instruments:

Guilt. From the Guilt Inventory [48], we used the subscale measuring guilt as a trait. The inventory contains 45 items divided into three subscales measuring moral beliefs, guilt as a trait, and guilt as a state. Example item: ‘I often have a strong feeling of regret’. Respondents answered on a five-step Likert scale ranging from (1) strongly agree to (5) strongly disagree. Items were coded so that higher numbers reflected greater guilt. Cronbach’s Alpha for the guilt scale is 0.87.

Shame. To measure shame we used the Experience of Shame Scale (ESS) [45], a 25-item questionnaire that assesses four areas of characterological shame: shame of personal habits, manner with others, type of person, and personal abilities; three areas of behavioral shame: shame of doing something wrong, saying something stupid, and failing in competitive situations; and body shame: feeling ashamed of one’s own body or any part of it. Item example: ‘Have you felt ashamed of your personal habits?’. Each item is rated on a 4-point scale from (1) not at all to (4) very much, giving total scores in the range 25–100. Cronbach’s Alpha for the shame scale is 0.96.

Parental Acceptance-Rejection. We used the short form of the Parental Acceptance-Rejection Questionnaire, child version (PARQ) [49], a self-report instrument designed to measure children’s perceptions of parental acceptance-rejection. The items are in the form ‘My mother doesn’t really love me’. Respondents answer such items on a 4-point Likert-type scale, from (4) almost always true to (1) almost never true. Scores range from 10 (minimum perceived undifferentiated rejection) to 40 (maximum perceived undifferentiated rejection). Scores equal to or greater than 25 indicate perceived undifferentiated rejection qualitatively greater than acceptance. For the group of participants in the present study, a Cronbach’s Alpha score of 0.95 was obtained for the mother questionnaire and 0.96 for the father questionnaire.

Abandonment. We used the Childhood Trauma Questionnaire (CTQ), [50], a questionnaire containing 28 items with responses on a Likert scale from (1) never true to (5) very often true. Item example: ‘My family members called me ‘stupid’, ‘lazy’ or ‘ugly’. For each of the five clinical scales—Emotional Abuse, Physical Abuse, Sexual Abuse, Emotional Neglect, and Physical Neglect—the five corresponding item scores are summed to obtain total scale scores ranging from 5 to 25 and provide a quantitative index of the severity of maltreatment experiences in each domain. For three items—minimization/denial—10, 16, 22—one point is added for each item reversed with a score of 5 (very often true). For each item reversed with a score of less than 5, no points are awarded. The total score ranges from 0 to 3. The scores of abuse were categorized into high and low based on the median. Cronbach’s Alpha for the total scale is 0.94.

### 5.6. Results 

#### 5.6.1. Correlation Matrix

Before testing the hypotheses, a multiple Pearson correlation analysis was performed between the main research variables in Jamovi.

Table 1 examines whether there is a relationship between the following variables: guilt, shame, physical and emotional neglect, sexual abuse, physical abuse, emotional abuse, paternal neglect, maternal neglect, and age. The resulting data indicated that out of 16 correlations, 11 were positive. Feelings of shame correlated positively with physical neglect, sexual abuse, physical abuse, and emotional abuse and negatively with emotional neglect, paternal rejection, maternal rejection, and age. Guilt correlates positively with emotional neglect, physical neglect, sexual abuse, physical abuse, emotional abuse, paternal rejection, and age, and negatively with maternal rejection. The largest correlation is between the emotional abuse variable and guilt, where the effect size is large (Cohen’s d = 1.186) [51]. The smallest correlation is between the paternal rejection and guilt variable, where the effect size is (Cohen’s d = 0.563). Of the 16 correlations, 18 correlations have a large effect size, 11 correlations have a medium effect size, and 5 have a small effect size. 

#### 5.6.2. Descriptive Statistics

**Figure 1:**
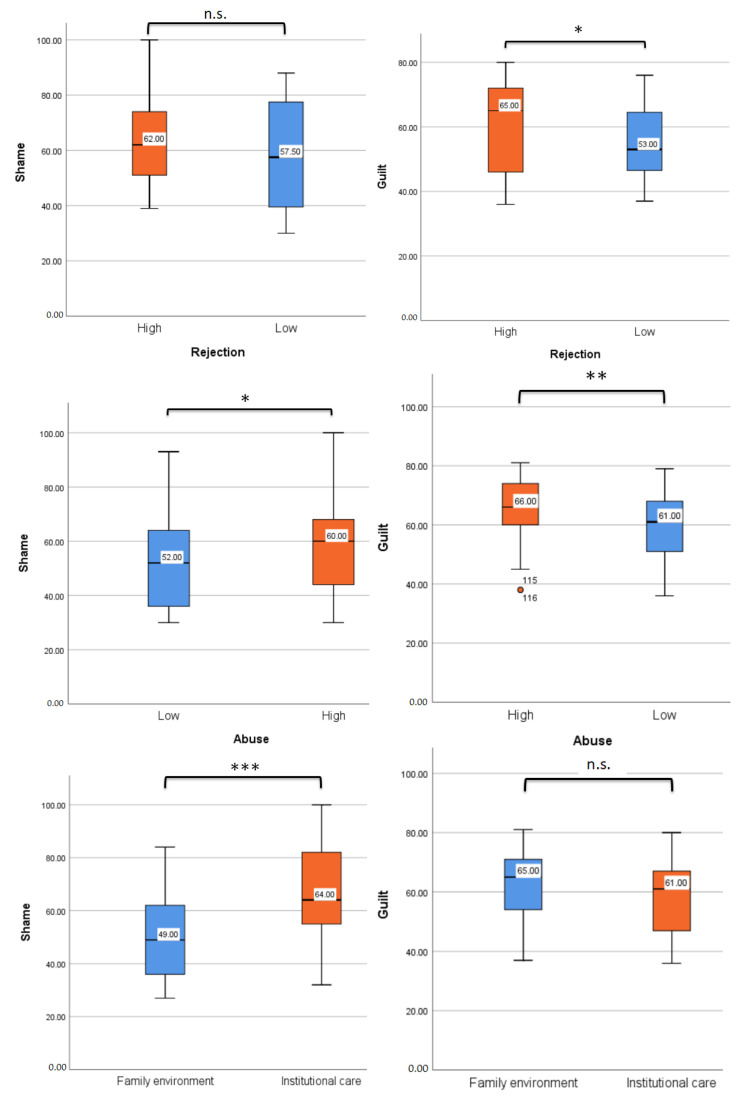
Boxplots for (1) comparing the distribution of Shame and Guilt scores of high and low rejection, (2) comparing the distribution of Shame and Guilt scores of low and high abuse, (3) comparing the distributions of the Shame and Guilt scores of the two groups of respondents. The boxplots present [1] the Lower Whisker (Q1–1.5 IR), [2] Lower Quartile or Q1, [3] Median (Q2), [4] Upper Quartile or Q3, [5] Upper Whisker Q3 + 1.5 IR. IR represents the Interquartile Range (Q3–Q1). The ‘*’ represent the outliers, which are single data points that are <1.5× the value of Lower Quartile. Notes: n.s. = not significant, * = denotes statistical significance at *p* < 0.05 level. ** = denotes statistical significance at *p* < 0.01 level. *** = denotes statistical significance at *p* < 0.001 level.

To provide descriptive data for the shame and guilt reports by abuse level, rejection level or living context, we performed a descriptive analysis (see Table 2 and Figure 1). 

#### 5.6.3. Hypotheses Testing

All our hypotheses were tested using independent samples *t* tests (see Table 2).

**Table 2:** The results of the independent samples *t* tests, for testing the hypotheses and means (*M*), standard deviations (*SD*), median (*Mdn.*) and the total number of participants (*N*).

Hypotheses	High Rejection	Low Rejection	*t*-Test	*df*	*p*
(H1) ‘Children and teenagers with higher levels of parental rejection will report stronger feelings of shame than those with lower levels of rejection.’	*N* = 38	*N* = 32	1.580	68.0	0.119
	*M* = 65.1	*M* = 58.0			
	*SD* = 17.4	*SD* = 20.1			
	*Mdn.* = 62.0	*Mdn.* = 57.5			
	*SE* = 2.830	*SE* = 3.567			
	High rejection	Low rejection			
(H2) ‘Children and teenagers with higher levels of parental rejection will report stronger feelings of guilt than those with lower levels of rejection.’	*N* = 38	*N* = 32	2.437	68.0	**0.017**
	*M* = 61.4	*M* = 54.2			
	*SD* = 12.8	*SD* = 11.7			
	*Mdn.* = 65.0	*Mdn.* = 53.0			
	*SE* = 2.07	*SE* = 2.08			
	High abuse	Low abuse			
(H3) ‘Children and teenagers with higher levels of abuse will report more shame than those with lower levels of abuse.’	*N* = 82	*N* = 74	−2.02	154	**0.045**
	*M* = 60.0	*M* = 54.1			
	*SD* = 18.0	*SD* = 18.8			
	*Mdn.* = 60.0	*Mdn.* = 52.0			
	*SE* = 1.98	*SE* = 2.18			
	High abuse	Low abuse			
(H4) ‘Children and teenagers with higher levels of abuse will report more guilt than those with lower levels of abuse.’	*N* = 74	*N* = 82	3.20	154	**0.002**
	*M* = 64.7	*M* = 58.8			
	*SD* = 11.2	*SD* = 11.7			
	*Mdn.* = 66.0	*Mdn.* = 61.0			
	*SE* = 1.31	*SE* = 1.30			
	Institutional care	Family environment			
(H5) ‘Children and teenagers raised in institutional care will report higher levels of shame than children raised in family environments.’	*N* = 58	*N* = 94	−5.27	150	**<0.001**
	*M* = 65.8	*M* = 50.6			
	*SD* = 19.0	*SD* = 16,1			
	*Mdn*. = 64.0	*Mdn.* = 49.0			
	*SE* = 2.50	*SE* = 1.66			
	Institutional care	Family environment			
(H6) ‘Children and teenagers raised in institutional care will report higher levels of guilt than children raised in family environments.’	*N* = 58	*N* = 94	1.06	150	0.289
	*M* = 60.2	*M* = 62.3			
	*SD* = 12.5	*SD* = 11.5			
	*Mdn*. = 61.0	*Mdn*. = 65.0			
	*SE* = 1.64	*SE* = 1.18			

Note: Bold values denote statistical significance at *p* < 0.05 level.

Participants with higher levels of parental rejection will indicate stronger feelings of shame than those with lower levels of rejection. The independent samples *t*-test indicated that there were no significant differences between children and teenagers with higher levels of rejection (*N* = 38, *M* = 65.1, *SD* = 17.4) and children and teenagers with lower levels of rejection (*N* = 32, *M* = 58.0, *SD* = 20.1): [t(68.0) = 1.580, *p* = 0.119, *p* > 0.05] in terms of feelings of shame. The effect size is small (Cohen’s *d* = 0.377). The hypothesis is disconfirmed.

Children and teenagers with higher levels of parental rejection will indicate stronger feelings of guilt than those with lower levels of rejection. The independent samples *t*-test indicated that there was a significant difference between participants with higher levels of rejection (*N* = 38, *M* = 61.4, *SD* = 12.8) and participants with lower levels of rejection (*N* = 32, *M* = 54.2, *SD* = 11.7): [t(68.0) = 2.437, *p* = 0.017, *p* < 0.05], in terms of feeling guilty. The effect size is medium (Cohen’s *d* = 0.587), and the hypothesis is confirmed.

To test whether children and teenagers with higher levels of abuse will show more shame than those with lower levels of abuse, we used the independent samples *t*-test, which indicated that there were significant differences between children and teenagers with higher levels of abuse (*N* = 82, *M* = 60.0, *SD* = 18.0) and children and teenagers with lower levels of abuse (*N* = 74, *M* = 54.1, *SD* = 18.8): [t(154) = −2.02, *p* = 0.045, *p* < 0.05], regarding the feelings of shame. The effect size is large (Cohen’s *d* = 1.515) and the hypothesis is confirmed.

Children and teenagers with higher levels of abuse will show more guilt than those with lower levels of abuse. The independent samples *t*-test indicated that there was a significant difference between children and teenagers with lower levels of abuse (*N* = 82, *M* = 58.8, *SD* = 11.7) and children and teenagers with higher levels of abuse (*N* = 74, *M* = 64.7, *SD* = 11.2): [t(154) = 3.20, *p* = 0.002, *p* < 0.05] in terms of guilt. The effect size is medium (Cohen’s *d* = 0.515), and the hypothesis is confirmed. 

To test whether children and teenagers raised in institutional care will show higher feelings of shame than children and teenagers raised in a family environment, we used the independent samples *t*-test. The results indicated that there was a significant difference between the level of shame in participants raised in an institutional environment (*N* = 58, *M* = 65.8, *SD* = 19.0) and the level of shame in participants raised in a family environment (*N* = 94, *M* = 50.6, *SD* = 16.1): [t(150) = −5.27, *p* < 0.001]. The effect size is large (Cohen’s *d* = 0.863), [52]. The hypothesis is confirmed.

To test whether there is a difference between the level of guilt experienced by children and teenagers raised in institutional care and children and teenagers raised in a family environment, we conducted an independent samples *t*-test. Results showed that there was no significant difference between the level of guilt in participants raised in institutional settings (*N* = 58, *M* = 60.2, *SD* = 12.5) and the level of guilt in children and teenagers raised in a family environment (*N* = 94, *M* = 62.3, *SD* = 11.5): [t(150) = 1.06, *p* = 0.289, *p* > 0.05]. This result indicates that there are no significant differences between groups. The effect size is trivial (Cohen’s *d* = 0.174). The hypothesis is thus disconfirmed.

## 6. Discussion

Regarding the results of hypothesis one, the association of shame with parental rejection is different from that of guilt; thus children and teenagers with higher levels of parental rejection experience shame to the same extent as respondents with lower levels of parental rejection. Some studies in the literature contradict this claim and argue that the relationship between parents and children greatly influences children’s sense of self and that growing up in an environment where parental rejection occurs has a direct impact on children’s sense of shame [27]. Other studies demonstrate that while the trauma of parental rejection does not necessarily generate feelings of shame, it can create a setting in which critically important standards of conduct have been violated by the parent [3]. Parental forms of maltreatment such as harsh parenting, sexual abuse, domestic violence and parenting characteristics during adolescence influenced adolescents’ proness to shame and guilt. Adolescents who experience harsh parenting or abuse may internalize feelings of shame and guilt, believing that they are responsible for the mistreatment they received. Witnessing domestic violence can also lead to feelings of shame and guilt as well as fear and anxiety. Furthermore, parenting characteristics during adolescence can also impact an individual’s proneness to shame and guilt. Parents who are overly critical or demanding may cause their children to feel inadequate or as if they can never meet their expectations. On the other hand, neglectful or dismissive parents may cause their children to feel unimportant or unworthy of attention, and children exposed to early psychosocial deprivation when they are brought up in institutional care can experience disruptions in typical brain development [52].

The results of hypothesis two indicate that the association of parental rejection with guilt is positive, with children and teenagers with higher levels of parental rejection experiencing different levels of guilt from those children and teenagers with lower levels of parental rejection. The higher the level of rejection, the more intense the guilt will be. This hypothesis is also supported by research [24]. Studies in the field have shown that those children who exhibit an anxious attachment style or experience feelings of non-acceptance and distrust as a consequence of parental rejection are more prone to more intense feelings of guilt [24].

The results of the third hypothesis indicate that abuse in all its forms is positively associated with feelings of shame. It seems that a high level of abuse is associated with stronger emotions of shame. The hypothesis is also supported by research suggesting that abused children and teenagers differ from non-abused respondents in terms of the emotions they express in relation to moral conflicts, especially in their feelings of shame [3]. Viewed as a traumatic experience, abandonment can increase children’s vulnerability to shame because they feel stigma as a result of abuse [53]. Abuse is associated with feelings of shame, psychological maltreatment behaviors, neglect, personal devaluation or parental rejection and indifference [3].

As for the association of abuse and guilt, according to hypothesis 4, a direct and positive link is observed. Thus, the more abuse, whatever its form, the greater the guilt. Another study [53] confirms this hypothesis by suggesting that abused children and teenagers differ from non-abused respondents in terms of moral emotions. The emergence of guilt is a reaction to the adversity to which a child is exposed. Studies [54,55,56] highlight that those children with negative experiences may develop a moral defense, thus accepting responsibility for the abuse they have suffered. In these circumstances, guilt is increasingly manifested, either because of the need for love and affection, needs that are denied or exploited, or because they ‘deserve bad treatment’ [56] (n.p.). 

The analysis of feelings of shame revealed that this is influenced by the respondents’ background. Children and teenagers from institutional care show a higher sense of shame than those in family settings. Previous research supports the idea that the family is the setting in which children and teenagers develop moral emotions. The experience of shame resulting from abandonment is caused by a lack of validation that compromises the sense of identity. Self-blame attributions and cognitions might be associated with feelings of shame [54].

According to the statistical results, feelings of guilt do not differ between children and teenagers growing up in institutional care and those growing up in a family environment. This indicates that the environment in which children and teenagers grow up does not have a significant impact on the manifestation of guilt. Although the result of the present study does not support this hypothesis, perhaps because the participants in this study who come from institutional care currently have better living conditions, other studies have shown that ‘children’s guilt has the potential to become maladaptive when children are exposed to chronically negative environments’ [52] (n.p.) and [57]. The authors also mention that ‘institutions, even in the best of circumstances, are characterized by suboptimal growth environments and psychosocial deprivation’ [52] (n.p.).

Finally, it would be positive to report that institutionalization, although the studies done on children raised in Eastern European orphanages are conclusive [58], does not always have to imply re-victimizing situations. Research demonstrates that attachment-based institutions can be healing for these abandoned children [59].

## 7. Conclusions

The present research aimed at investigating whether abandonment influences adolescents’ feelings of shame and guilt. Abandonment is viewed in this paper through the lens of parental abuse, neglect, and rejection. The results showed: (1) children and teenagers growing up in institutional care report higher levels of shame than children and teenagers growing up in family settings, but there are no differences in guilt; (2) the higher the level of abuse, the more intense the feelings of shame and guilt; (3) feelings of guilt but not shame are influenced by parental rejection; (4) the stage of adolescence is not a factor in increasing feelings of shame and guilt.

The environment in which children and teenagers grow up, whether in a family or in an institution, matters when it comes to the emergence of feelings of shame and guilt. The parent or parental figures may shape these moral emotions and they can induce feelings of guilt and shame in their children [60,61,62,63]. The results of our research are additional clues that highlight the association between childhood trauma and feelings of shame and guilt.

A limitation of this study is the small number of respondents. Another limitation is the disproportionate size of the two groups: there was a smaller number of participants in the institutional care system group than in the family environment group. Furthermore, the study was based on self-reported data, which raises concerns about the potential bias in giving socially desirable responses. 

It is also important to consider various other factors when assessing the level of shame or guilt experienced by an individual. These factors include the nature of their relationship with their biological family, the age at which they were abandoned, the number of institutions they have been in, whether they have maintained a relationship with their biological family, their connections with extended biological family members (if any), and whether they have been through an adoption process, among others. That these variables were not considered in the present study is a limition, but by taking these variables into account, future studies can gain a more comprehensive understanding of the individual’s experiences and emotions, which represents a research opportunity. Future studies can also investigate how different types of child-protection measures are associated with emotions of shame and guilt.

Adolescents from abusive or broken homes may, in some cases, resort to self-medicating behaviors such as alcohol or drug abuse, engaging in sexual activities with siblings who cannot give true consent, or threatening and harming siblings, parents, or even law enforcement officials with knives or other weapons. These actions can lead to feelings of guilt and shame, compounding the already challenging circumstances these young individuals face. It is crucial for future research to consider these variables when examining the effects of abuse and broken homes on adolescent behavior. By doing so, we can gain a more comprehensive understanding of the complex and often devastating consequences of these experiences.

Institutionalized abandoned children who have high levels of shame and guilt can be helped in different ways. Probably the first approach is to provide a supportive and nurturing environment within the institution itself. This can include hiring staff who are trained in trauma-informed care, creating a sense of community among the children, and providing opportunities for positive experiences such as art therapy or outdoor activities. It is also important to address the root causes of abandonment and institutionalization, such as poverty, neglect, or abuse. This may involve working with families or communities to provide resources and support that can prevent children from being abandoned in the first place. Furthermore, these children can be helped by helping them understand their stories, teaching them and encouraging them to communicate openly about their personal problems, or fostering a sense of belonging and of purpose in life. Ultimately, helping institutionalized abandoned children who have high levels of shame and guilt requires a multifaceted approach that addresses both their emotional needs and the systemic issues that contribute to their situation.

## Figures and Tables

**Table 1:** Descriptive Statistics and Correlations for Study Variables.

		S	G	PN	EN	SA	PA	EA	PR	MR
S	*M* = 57.0 *SD* = 18.7 *N* = 230	-								
G	*M* = 61.6 *SD* = 11.8 *N* = 230	−0.496 **	-							
PN	*M* = 8.4 *SD* = 3.8 *N* = 226	0.347 **	−0.415 **	-						
EN	*M* = 10.6 *SD* = 5.1 *N* = 226	0.146	−0.228 **	0.39 **	-					
SA	*M* = 6.04 *SD* = 3.04 *N* = 226	0.288 **	−0.360 **	0.575 **	0.373 ***	-				
PA	*M* = 7.6 *SD* = 4.1 *N* = 226	0.332 **	−0.400 **	0.31 **	0.509 ***	0.611 **	-			
EA	*M* = 10.2 *SD* = 5.4 *N* = 226	0.474 **	−0.510 **	0.532 **	0.686 ***	0.489 **	0.750 **	-		
PR	*M* = 18.6 *SD* = 15.7 *N* = 220	0.030	0.271 *	0.292 ***	0.397 **	0.240 **	0.302 **	0.454 **	-	
MR	*M* = 13.8 *SD* = 12.4 *N* = 230	0.095	0.186	0.309 **	0.494 ***	0.089	0.278 **	0.566 **	0.463 ***	-
A	*M* = 17.1 *SD* = 1.82 *N* = 230	−0.224 **	0.072	0.295 **	0.198 **	−0.091	−0.095	−0.068	0.068	0.068

Note: (1) S—shame; G—guilt; PN—physical neglect; EN—emotional neglect; PA—physical abuse; SA—sexual abuse; EA—emotional abuse; PR—paternal rejection; MR—maternal rejection; A—age. (2) * *p* < 0.05, ** *p* < 0.01, *** *p* < 0.001.

## Data Availability

Not applicable.
